# Development of thymic tumor in [*LSL:Kras*^*G12D*^; *Pdx1-CRE*] mice, an adverse effect associated with accelerated pancreatic carcinogenesis

**DOI:** 10.1038/s41598-021-94566-x

**Published:** 2021-07-23

**Authors:** Sophie Liot, Naïma El Kholti, Jonathan Balas, Laurent Genestier, Bernard Verrier, Ulrich Valcourt, Elise Lambert

**Affiliations:** 1 Laboratoire de Biologie Tissulaire et Ingénierie Thérapeutique (LBTI), UMR CNRS 5305, Université Claude Bernard Lyon 1, Institut de Biologie et Chimie Des Protéines, 7, passage du Vercors, 69367 Lyon Cedex 07, France; 2 UR LIB « Lymphoma Immuno-Biology”, Université Claude Bernard Lyon I, Lyon, France

**Keywords:** Cancer models, Cancer, Cell biology

## Abstract

Pancreatic Ductal AdenoCarcinoma (PDAC) represents about 90% of pancreatic cancers. It is one of the most aggressive cancer, with a 5-year survival rate below 10% due to late diagnosis and poor therapeutic efficiency. This bad prognosis thus encourages intense research in order to better understand PDAC pathogenesis and molecular basis leading to the development of innovative therapeutic strategies. This research frequently involves the KC (LSL:*Kras*^*G12D*^;*Pdx1*-CRE) genetically engineered mouse model, which leads to pancreatic cancer predisposition. However, as frequently encountered in animal models, the KC mouse model also exhibits biases. Herein, we report a new adverse effect of *Kras*^*G12D*^ mutation in KC mouse model. In our hands, 10% of KC mice developed clinical signs reaching pre-defined end-points between 100- and 150-days post-parturition, and associated with large thymic mass development. Histological and genetic analyses of this massive thymus enabled us (1) to characterize it as a highly proliferative thymic lymphoma and (2) to detect the unexpected recombination of the Lox-STOP-Lox cassette upstream *Kras*^*G12D*^ allele and subsequent KRAS^G12D^ protein expression in all cells composing thymic masses. Finally, we highlighted that development of such thymic tumor was associated with accelerated pancreatic carcinogenesis, immune compartment disorganization, and in some cases, lung malignancies.

## Introduction

Pancreatic cancer, among which Pancreatic Ductal AdenoCarcinoma (PDAC) represents about 90% of cases, is currently the seventh cause of cancer-related deaths worldwide, and is predicted to become second in industrialized countries over the next decade if no progress is made^1–3^. PDAC is one of the most aggressive cancer, with a 5-year survival rate below 10%, due to late diagnosis and poor therapeutic efficiency^4^. Indeed, PDAC develops without specific symptoms, leading to diagnosis at locally advanced or metastatic tumor stage^5,6^. As a consequence, only 20% of PDAC are resectable, and classical chemotherapies are inefficient, only prolonging patient lifespan by a few months^7^. Thus, PDAC is currently the subject of intense research to develop innovative therapeutic strategies, based on the understanding of PDAC pathogenesis and molecular basis.

Fundamental research in the field of oncology is based on the use of models recapitulating human pathology. In the case of PDAC, a large spectrum of models is available, including, from the farthest to the closest of human pathogenesis, 2D and 3D (organotypics) in vitro cell culture systems with tumor cell lines, organoids and animal models^8,9^. These latter are now widely used and comprise several experimental strategies. Historically, PDAC was chemically induced by the injection of potent carcinogens such as DMBA (7,12-DiMethylBenz(a)Anthracene) in the pancreas. This method has been abandoned in favor of xenografts (of tissues or cell lines derived from patients) and genetically-engineered mouse models (GEMM)^10,11^, which can be considered to recapitulate as close as possible the carcinogenesis, invasive tumor progression and metastasis formation of PDAC. GEMM notably allow to study risk factors, genetic alterations, as well as PDAC initiation and progression, and represent a pre-clinical study platform for the development of new diagnosis methods and treatments^12–15^.

First GEMM used in PDAC research were established by the overexpression of Transforming Growth Factor (TGF)α under the control of *Elastase* promoter (specific of pancreatic acinar cells)^16,17^. However, this model did not allow to trigger invasive PDAC and was then replaced by several models involving mutated *Kras* oncogene. KRAS (for Kristen RAS) is a small GTPase of the RAS family having a preponderant role in malignancies, since it is involved in several signaling pathways leading to the activation of cell proliferation^18–20^. Single KRAS amino-acid mutation, often on residue Gly^12^, results in KRAS constitutive activation and subsequent cell behavior modification. *KRAS* proto-oncogene mutations have been detected in about 30% of human tumors^21^, and 92% of PDAC, where they correlate with a worse prognosis^22^. In PDAC, *KRAS* mutation is even considered as the first hit mutation (detected from the pre-malignant stages), leading to cell sensitivity to malignant transformation induced by the accumulation of other mutations (notably the inactivation of tumor suppressor genes *TP53*, *CDKN2A* (also named *INK4A*) and *SMAD4*) and chromosomal aberrations.

As a consequence, *Kras* mutation in mouse results in the development of malignancies^23^. In 2001, Jackson and colleagues developed a mouse model allowing to induce conditional KRAS activation thanks to the CRE-Lox system, with the mutated *Kras*^G12D^ oncogene placed downstream a Lox-STOP-Lox sequence (transcriptional STOP)^24^. This construct has then been used in 2003 in the same laboratory to create a pancreatic cancer predisposition model, in which the CRE recombinase expression is under the control of pancreas-specific gene promoters: *Pdx1* or *Ptf1a* (*p48*), allowing a tissue-specific expression of *Kras*^G12D^ (model named “KC”, for [LSL:*Kras*^G12D^*;Pdx1* or *Ptf1a*-CRE])^25^. These mice develop pancreatic preneoplastic lesions similar to human ones, but rarely evolving into PDAC. In order to reduce this long latency limiting PDAC progression study, other GEMM have been established with additional mutations in the [LSL:*Kras*^*G*12*D*^*;Pdx1*-CRE] context, including *p53* (“KPC model”)^26^ and *Ink4a* (“KIC model”)^27^ mutated tumor suppressor genes, enabling the development of invasive PDAC within few months^28^.

Nowadays, KC mouse model (which will refer to [LSL:*Kras*^*G*12*D*^*;Pdx1*-CRE] from now) continues to be widely used, as it allows a good understanding of pancreatic pre-neoplastic lesions preceding invasive PDAC. Indeed, this model recapitulates the main characteristics of human pancreatic carcinogenesis, including desmoplastic reaction and PanIN linear development. It thus provides a better understanding of the key signaling pathways involved in these processes and is a suitable model for the search of an early diagnostic method^14^. However, it is still important to keep in mind that GEMM do not totally recapitulate human pathogenesis and may have some undesirable effects, and as such, experimental limitations and biases have been reported for the KC model. Indeed, Pdx1 (Pancreatic and duodenal homeobox 1) is a transcription factor activating the expression of various pancreatic proteins, such as insulin, and is highly involved in pancreas development, as this developmental factor is responsible for pancreatic identity induction^29,30^. As a consequence, KC model is a “pre-natal” model, in which *Kras*^*G*12*D*^ mutated oncogene will be expressed during early steps of pancreatic development, and thus does not exactly mimic the human pathogenesis^28,31,32^. Furthermore, at adulthood, *Pdx1* is classically expressed in duodenum and pancreas. In this latter, it has mainly been detected in nuclei of β cells (endocrine cells of Langerhans islets) and to a lesser extent in the nuclei of ductal and acinar cells^33,34^. Therefore, it is not possible to decipher the cells that are at the origin of PDAC using this model, since *Kras*^*G*12*D*^ will be expressed in the different pancreatic epithelial cells. Additionally, islet disorganization as well as lesions within the endocrine part of the pancreas have also been highlighted^35,36^, showing some limitations of this model.

Herein, we reported a new adverse effect of *Kras*^*G*12*D*^ mutation in the KC mouse model, which has not yet been described. In our hands, between 100- and 150-days post-parturition, 10% of the KC mice developed clinical signs corresponding to pre-defined humane end-points, including weight loss and respiratory distress. At necropsy, all concerned mice presented a large thymic mass filling the entire space inside the rib cage, crushing the lungs, heart and esophagus. Through histological and immunohistochemical analyses, we demonstrate that this thymic mass corresponds to a highly proliferative thymic lymphoma. In all concerned thymus, we detected the recombination of the Lox-STOP-Lox cassette upstream *Kras*^*G*12*D*^ allele and subsequent KRAS^G12D^ protein expression in all thymic mass cells, as opposed to a normal thymus. In addition, we highlighted that development of such a thymic tumor was associated with accelerated pancreatic carcinogenesis and immune compartment disorganization (visible in the spleen), and observed some mice with lung malignancies. Our study thus alerts about the presence of a non-silent side effect of *Kras*^*G*12*D*^ mutation in 10% of KC mice, which may be missed if dissection is restricted to the abdominal cavity.

## Results

### In KC mouse model, LSL recombination occurs in pancreas but not in tail

**Figure 1:**
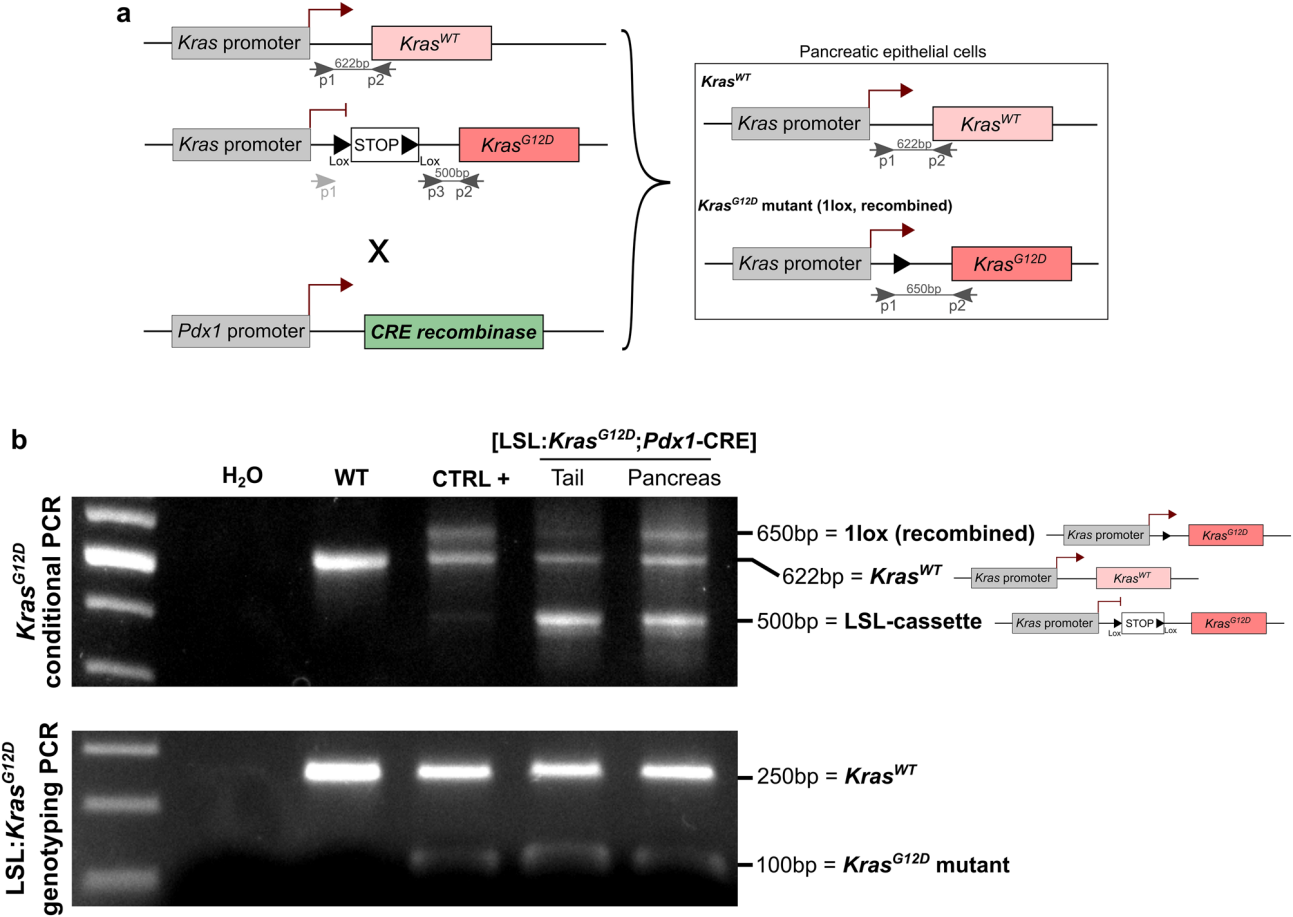
KC (LSL:*Kras*^G12D^:*Pdx1*-CRE) mouse model. (**a**) Schematic representation of the genetic construct for conditional *Kras*^G12D^ expression under the control of *Pdx1* promoter (CRE-Lox system). Primers used for *Kras*^G12D^ conditional PCR are noticed. (**b**) Top: *Kras*^G12D^ conditional PCR showing LSL cassette recombination in pancreas, but not tail, from KC mouse, and not in WT mouse. Bottom: *Kras*^G12D^ genotyping PCR showing heterozygosity in KC mice (WT and *Kras*^G12D^ alleles) and homozygosity in WT mouse (WT allele). Uncropped gels are provided in Supplementary Figure S2.

KC mouse model were obtained by crossing the *Pdx1*-CRE and LSL:*Kras*^*G*12*D*^ mouse strains, in order to study pancreatic carcinogenesis. In the LSL:*Kras*^*G*12*D*^ strain, the endogenous *Kras*^*G*12*D*^ allele is preceded by a Lox-STOP-Lox (LSL) cassette avoiding *Kras*^*G*12*D*^ transcription in absence of recombination. In order to induce a conditional expression of the mutated oncogene in the pancreas, we used the *Pdx1*-CRE mouse strain, in which the CRE recombinase coding sequence is placed downstream of the *Pdx1* gene promoter (Fig. 1a).

Mice were genotyped one-week post-parturition by a classical Polymerase Chain Reaction (PCR). All KC mice carried both the wild-type (WT) and the mutated (LSL:*Kras*^G12D^) *Kras* alleles, while WT mice presented only the WT *Kras* band (Fig. 1b, bottom). In order to confirm correct pancreas-specific recombination, conditional *Kras*^*G12D*^ PCR was performed on tail and pancreas of one KC mouse (Fig. 1b, top). Primers used for this PCR, which allows to detect LSL cassette recombination, are presented in Fig. 1b. In tail of KC mice, we observed wild-type (WT) and LSL:*Kras*^*G12D*^ bands only, while in pancreas we noted the presence of a third band (corresponding to *Kras*^G12D^ without the LSL cassette). The coexistence of the 3 bands in pancreas suggests that recombination is partial, not in all cells composing the tissue extract. As controls, we analyzed pancreatic DNA from (1) WT mouse pancreas, for which we detected the WT *Kras* allele only, with a large band, and (2) KC mouse tumor tissue with almost total recombination (CTRL+), which showed mainly the presence of the WT and *Kras*^G12D^ alleles without the LSL cassette. This result confirmed the correct pancreas-specific recombination of mutated *Kras*^G12D^ oncogene.

### 10% of KC mice display severe clinical signs associated with thymic mass development

**Figure 2:**
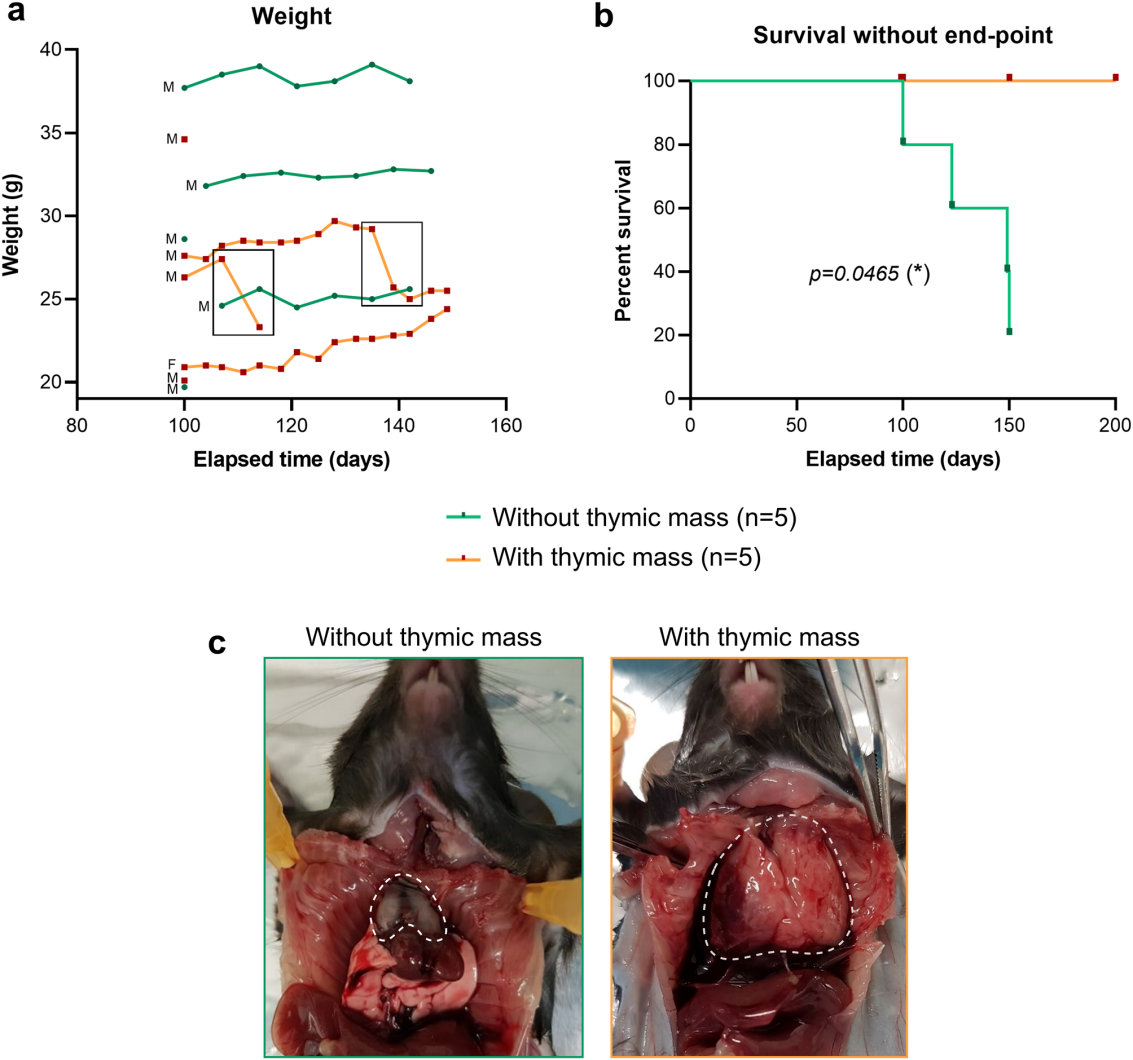
**10% of KC mice exhibited rapid weight loss and decreased survival associated with thymic mass development. **(**a**) Body weight monitoring of the KC mice presented in this study. Sex of the different mice is indicated on the left (M = male, F = female) (**b**) Cumulative survival proportion of the two groups presenting (orange, n = 5) or not (green, n = 5) a thymic mass, estimated using the Kaplan–Meier method. The two groups were compared thanks to Log-Rank (Mantel-Cox) statistical test. (**c**) Photo of the rib cage at autopsy, showing normal thymus (left) and the thymic mass developed in 10% of KC mice (right). Dotted line delimits thymus. Scale bars are indicated on the pictures.

While constituting a cohort of KC mice invalidated or not for our gene of interest, we unexpectedly observed some KC mice suffering from severe clinical signs between 100- and 150-days post-parturition independently from this gene of interest. Those clinical signs included drastic weight loss and/or respiratory distress, and corresponded to pre-defined end-points, thus leading us to euthanize the animals before the scheduled date. These mice finally accounted for about 10% of the total cohort (9/89), which thus cannot be ignored. In the work presented here, we decided to characterize the 5 KC mice (4 males and 1 female) displaying such suffering signs in our cohort that we compared to 4 age-matched KC mice (100 and 150 days) and 1 older mouse (200 days), without apparent symptoms from the same cohort. Body weight monitoring has been performed on mice from 100 days post parturition, and show the rapid weight loss of two mice with thymic tumors (Fig. 2a). It also has to be noted that we observe a high body weight variation between each mouse of the two groups, that cannot be due to the sex of the mice since they are mostly males, but rather to the fact that the mice are mostly not littermates. Finally, we calculated cumulative survival proportion depending on the development or not of thymic mass (Kaplan–Meier method, Fig. 2b), and clearly showed that mice harboring thymic mass had poorer overall survival compared to unaffected mice, coherently with the observed end-points and subsequent euthanasia. During dissection, we observed that all mice reaching a humane end-point had a rib cage invaded by a huge mass clearly developed from thymus. This thymic mass caused the crushing of esophagus, heart and lung, explaining the observed clinical signs (Fig. 2c). It has to be noted that in the Fig. 2c, lung and heart were clearly visible in the mouse with a histologically normal thymus (left photo), but completely hidden by the thymic mass in the symptomatic mouse (right photo).

### Pathological thymus exhibits total LSL cassette recombination, with subsequent KRAS^*G12D*^ expression and thymic lymphoma development

**Figure 3:**
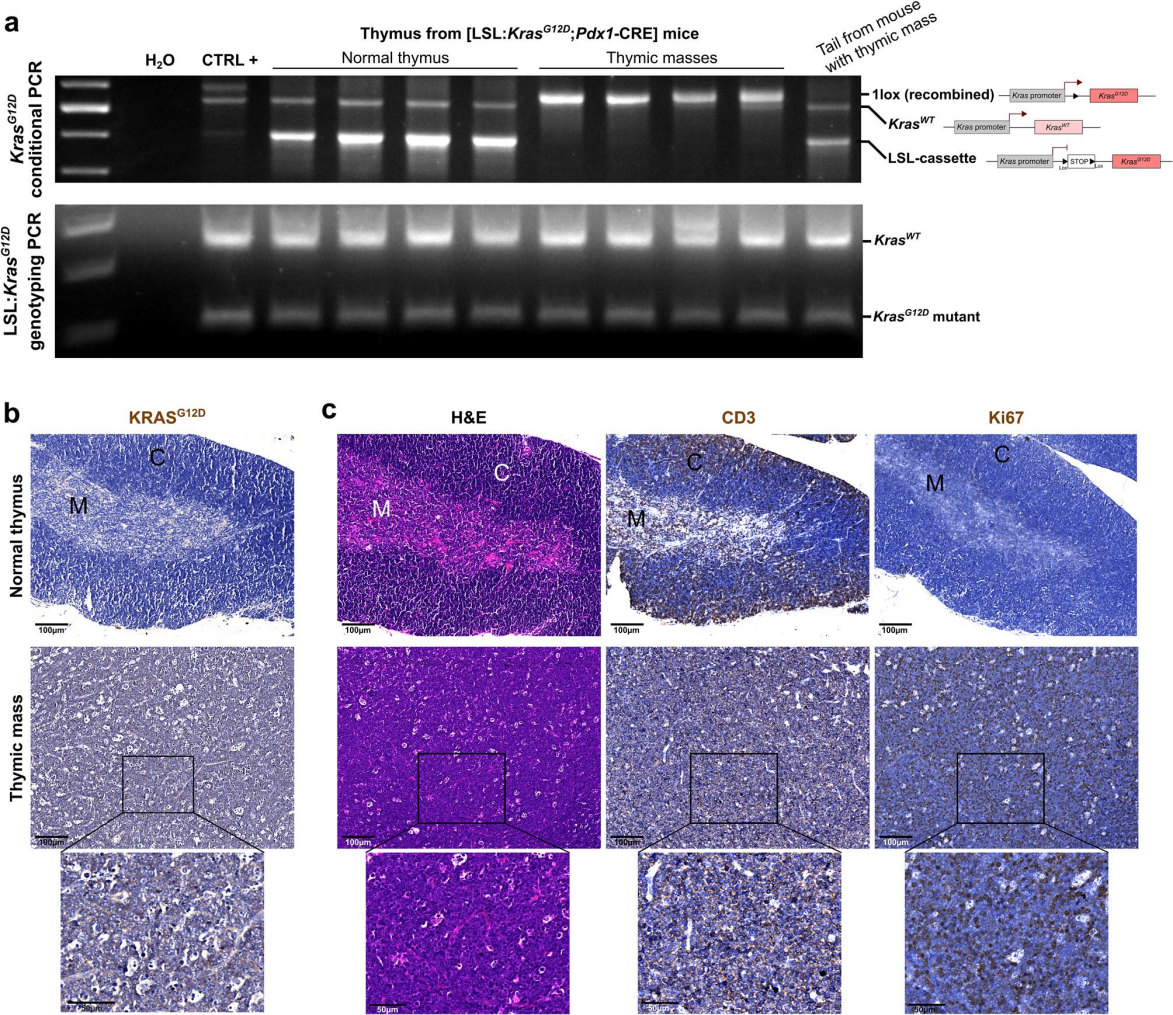
**Thymic mass showed a lymphoma-like histology and total LSL cassette recombination associated with KRAS **^G12D^** expression in all cells.** (**a**) Top: *Kras*^G12D^ conditional PCR showing LSL cassette recombination in thymic mass but not in normal thymus and tail (KC mice). Bottom: *Kras*^G12D^ genotyping PCR showing heterozygosity in all KC mice of this study (WT and *Kras*^G12D^ alleles). Uncropped gels are provided in Supplementary Figure S2. (**b**) Representative images of normal thymus and thymic mass immunolabelled for KRAS^G12D^. (**c**) Representative images of normal thymus and thymic mass histologically analyzed (H&E staining and CD3e and Ki67 immunohistochemical labelings). C = cortex, M = medulla. Scale bars are indicated on the pictures.

We then suspected an artefactual recombination of LSL cassette in the developing thymic masses and consequently performed a conditional *Kras*^*G12D*^ PCR on these tissues, (Fig. 3a, upper panel). Interestingly, we highlighted that normal thymus did not present any LSL cassette recombination, while the recombination was total in thymic masses. Indeed, in those tissues, we were not able to detect any LSL:*Kras*^G12D^ band, suggesting that all cells proceeded to LSL cassette recombination and thus expressed *Kras*^G12D^ allele. Moreover, such recombination seemed to be specific to the thymus, as no recombination of the LSL cassette was detected in the tail of mice suffering from thymic mass development. It has to be noted that WT *Kras* amplicon was always visible in normal thymus, while it was sometimes difficult to discriminate in thymic masses. Genotyping PCR was performed on tail from all analyzed animals to validate the heterozygosity of the *Kras*^*G12D*^ allele for all KC mice (homozygous *Kras*^*G12D*^ is lethal) (Fig. 3a, lower panel). Finally, we histologically confirmed the consequence of the LSL cassette excision on paraffin-embedded thymus sections, by showing that all cells expressed KRAS^G12D^ in thymic mass, while none were positive in normal thymus (Fig. 3b).

To better characterize the consequence of such artefactual recombination and KRAS^G12D^ expression in thymus, we further analyzed these thymic masses and compared them to normal thymus. Paraffin-embedded tissues were cut and sections were stained with Hematoxylin–Eosin or labelled by immunohistochemistry (IHC) for CD3e (T lymphocytes) and Ki67 (proliferation) (Fig. 3c). As expected, normal thymus was composed of (1) a connective tissue capsule, (2) the cortex (C), which is darker and contains lymphocytes (CD3e-positive cells) and (3) the medulla (M), containing larger lymphocytes (CD3e-positive cells) and Hassall’s Corpuscles. In contrast, the thymic masses did not show such organization, and were very rich in lymphocytes (round cells predominantly occupied by the nucleus), reminiscent of a thymic lymphoma. This was confirmed by Ki67 and CD3e immunolabelings of the thymus demonstrating highly proliferative cells, positive for lymphocytic marker (Fig. 3c).

Therefore, LSL cassette recombination and subsequent mutated *Kras*^*G12D*^ oncogene expression occurred in thymus of 10% of KC mice, leading to the development of thymic lymphoma**.**

### Thymic lymphoma development is associated with accelerated pancreatic carcinogenesis, lung malignancies and immune disorder

**Figure 4:**
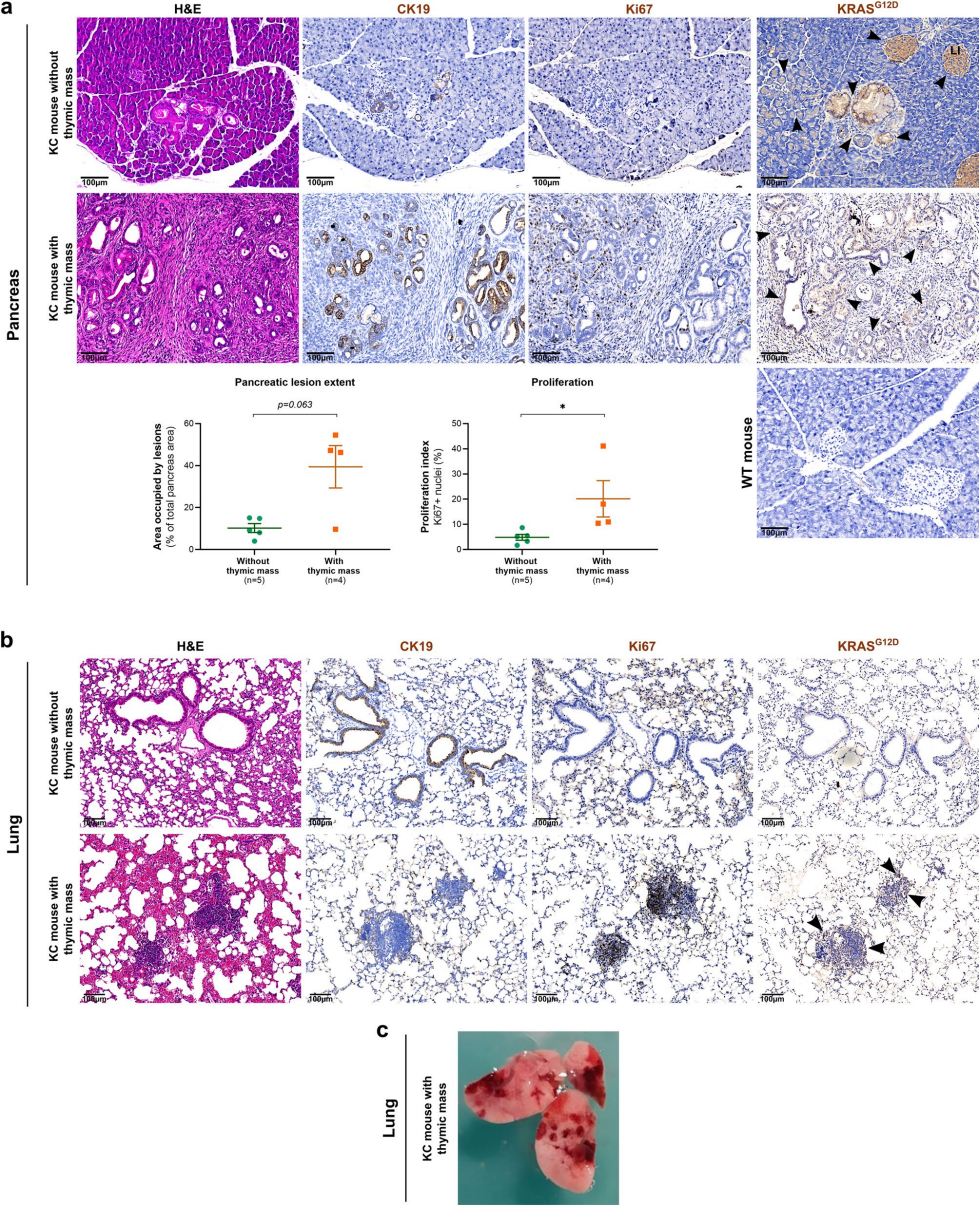
**KC mice developing thymic tumor exhibited accelerated pancreatic carcinogenesis, immune perturbations and lung malignancies.** (**a**) Representative images of pancreas from KC mice showing or not thymic mass analyzed by classical histology (H&E staining) and immunohistochemistry (Ki67, CK19, and KRAS^G12D^). Quantification of the area occupied by lesions in pancreas sections and of proliferating cells (Ki67-positive nuclei) are presented below the pictures. Groups were compared thanks to Mann–Whitney statistical test (non-parametric *t *test). (**b**) Representative images of histological analysis of lung from KC mice with or without thymic mass (H&E staining and Ki67, CK19 and KRAS^G12D^ immunohistochemistry). Bottom: This mouse displayed moderate lesions in lung. (**c**) Picture showing severe lung lesions, macroscopically visible, from KC mouse presenting thymic mass at autopsy. (**a,b,d**) Arrowheads highlight KRAS^G12D^ staining. Scale bars are indicated on the pictures.

Finally, we wanted to decipher if such thymic tumor development could have an effect on pancreatic carcinogenesis. We thus analyzed the pancreas of mice exhibiting or not a thymic mass, by classical H&E staining and CK19 (pancreatic ductal cell/malignant epithelial carcinoma marker) and Ki67 immunolabelings (Fig. 4a). Lesion extent (area occupied by lesions visible in H&E staining) and proportion of proliferating cells within lesions have been quantified thanks to QuPath software. We clearly noticed that mice with thymic tumor displayed larger area occupied by pre-tumoral lesions in three of the four mice (Fig. 4a, left). The difference between the two groups is not significant, most likely due to low number of mice and the fact that one of the mice with thymic tumor did not present larger lesion extent yet. Nevertheless, we were able to detect a significant increase in proliferation, with more Ki67-positive cells in pancreatic lesions of mice suffering from thymic tumor, allowing us to affirm that mice displaying thymic tumors presented more aggressive lesions.

In order to better understand KRAS^G12D^ expression in KC mouse model, we immunolabelled pancreas and detected KRAS^G12D^ mutated protein within preneoplastic lesions, as well as in Langerhans islets of all KC mouse pancreas (with and without thymic lymphoma), in contrast to WT mouse, which did not display any labeling. In addition, KRAS^G12D^ was detected in acinar cells bordering pre-tumoral lesions, and that seemed to undergo Acinar-to-Ductal Metaplasia (ADM). In mice suffering from thymic lymphoma, KRAS^G12D^ labeling area was more important, in view of the increased extent of lesions in their pancreas (Fig. 4a, right).

Interestingly, in three out of four mice exhibiting thymic mass development, we also noticed proliferative (Ki67-positive) masses in lungs, which were also positive for KRAS^G12D^ (Fig. 4b). These lung malignancies were negative for CK19 and did not show any PDAC morphology. In one of them, such masses were even visible macroscopically at necropsy (Fig. 4c).

**Figure 5:**
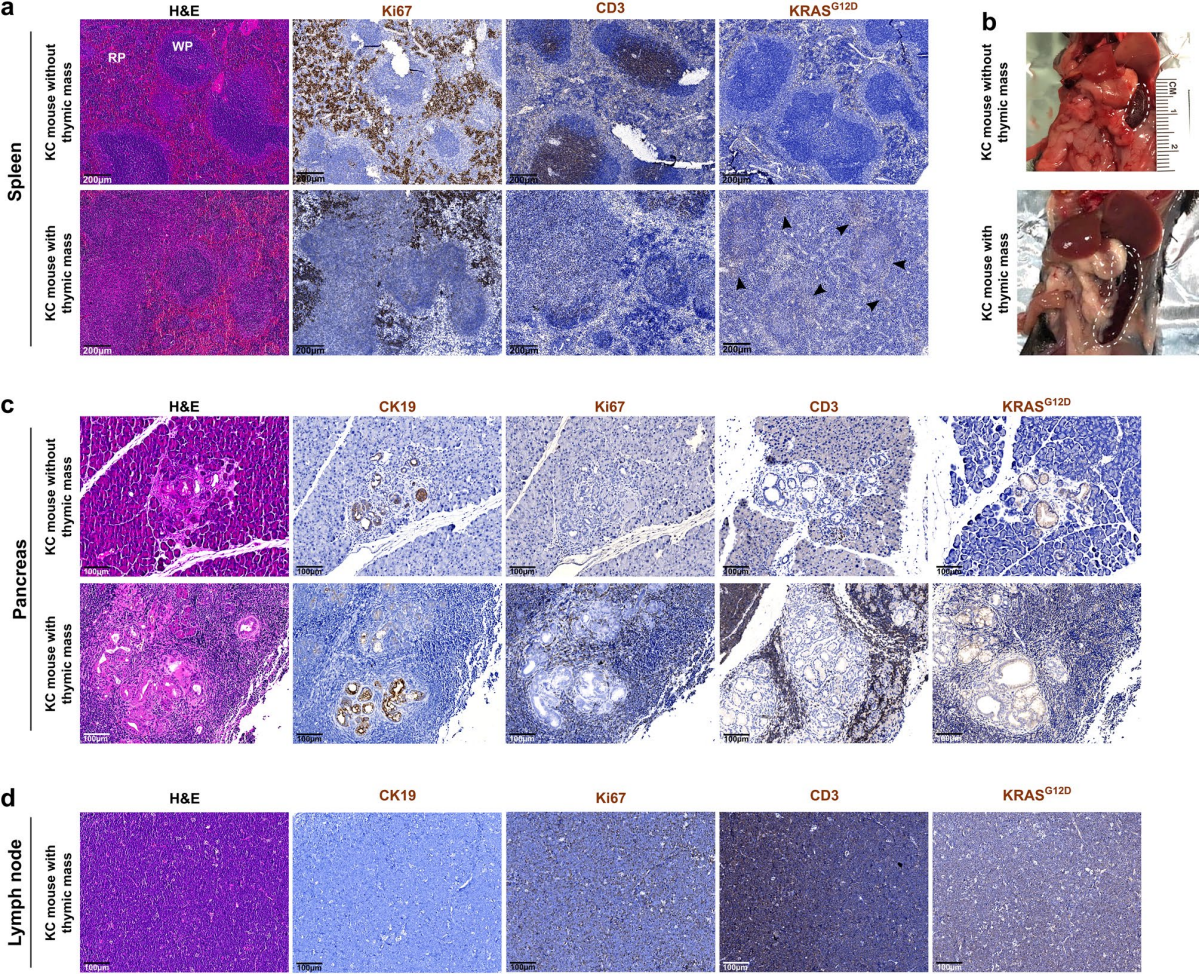
**Immune dysregulation in mice suffering from thymic lymphoma development.** (**a**) Representative images of histological analysis of spleen from KC mice presenting or not thymic mass (H&E staining and Ki67 and KRAS^G12D^ immunohistochemistry). (**b**) Image showing enlarged spleen from KC mouse with thymic mass. (**c**) Representative images of histological analysis of the pancreas of KC mouse without thymic lymphoma, and one of the two mice with thymic mass which presented high level of immune infiltration (H&E staining and CK19, Ki67, CD3e and KRAS^G12D^ immunolabelings). (**d**) Representative images of histological analysis of the proximal lymph node of one of the two mice with thymic mass which presented high level of immune infiltration (H&E staining and CK19, Ki67, CD3e and KRAS^G12D^ immunolabelings). Scale bars are indicated on the pictures.

Thymus being one of the primary lymphoid organs, we also investigated the immune compartment, notably thanks to spleen analysis. Interestingly, mice with thymic tumor displayed highly enlarged spleen, which seemed to be histologically disorganized (Fig. 5a,b). Indeed, white pulp seemed larger, and less organized and dense, as it can be observed following H&E staining and Ki67 and CD3e immunolabelings. In addition, KRAS^G12D^ protein was detected in white pulp of spleen from mice developing thymic mass, suggesting either the recruitment of lymphocytes from the thymus or recombination of the LSL cassette within the spleen too. In addition, two of the thymic tumor-suffering mice also showed an important immune infiltration. Indeed, in the pancreas of those mice, numerous highly proliferating immune cells were observed, not only around lesions but also around still normal acini, as showed by CD3e (T lymphocytes) and Ki67 (proliferation) immunolabelings (Fig. 5c). In addition, this was accompanied by the presence of large lymph nodes on section (Fig. 5d). Those immune cells observed in close proximity to pancreas were also KRAS^G12D^-positive, evoking recruitment of lymphocytes from pathological thymus. Both results delineate immune dysregulation within mice suffering from thymic mass development.

## Discussion

Taken together, our results highlight a new adverse effect linked to genetic modification in the KC mouse model. Indeed, we pointed out the development of thymic tumor in 10% of the KC mouse cohort, which cannot be ignored. Interestingly, our observations were made in two different animal facilities, indicating that it would better rely on intrinsic mechanism than on environmental cues. Mice presenting thymic mass reached humane end-points between 100- and 150-days post-parturition, including rapid weight loss and respiratory distress, probably due to lung, heart and esophagus crushing under the tumor mass, since these organs were smaller than in normal KC mice. In addition, those mice were prostrated in their cage, suggesting abdominal pain. Such symptoms are close to the ones developed in consequence to PDAC development and are thus confounding for this mouse model at early age. It must be noted that presence of this thymic mass could not be predicted without opening rib cage, showing the importance to proceed to a complete necropsy.

We first investigated *Kras*^G12D^ expression within these thymic masses. To do so, we first analyzed LSL cassette recombination upstream *Kras*^G12D^ allele and showed that the transcriptional STOP was deleted in all thymic masses, while it was never the case in normal thymus. Interestingly, this recombination was total, with no remaining allele with the LSL cassette, suggesting that all cells would have recombined, in contrast to pancreas where the LSL sequence persisted. It is important to note that LSL cassette recombination is not general in the mice harboring thymic tumor; since it is not recombined in tail. This was confirmed by anti-KRAS^G12D^ immunohistochemistry, as we detected labeling in all cells from thymic masses, and not in normal thymus. Histologically, thymic masses showed a disorganized structure, with the absence of distinguishable cortex and medulla, in contrast to normal thymus. Tumors appeared to be lymphocyte-rich and highly proliferative, evoking thymic lymphoma, with few epithelial cells^37–39^. This diagnosis is further reinforced by the strong similarities observed at the macroscopic and microscopic levels between the pathological thymus of KC mice and the thymic lymphoma developed in the p53 KO mice^40^. Thus, we can confidently hypothesize that the development of thymic tumors, would rely on the expression of mutated *KRAS* oncogene.

Initially, our hypothesis for such atypical *Kras*^*G12D*^ expression was that *Pdx1* could be transiently expressed by thymic antigen-presenting cells for lymphocyte education, with subsequent *Kras*^G12D^ expression, which could lead to malignant transformation in some cases. However, the described observations suggest another mechanism. The lymphoma phenotype of thymic tumoral mass, as well as the expression of *Kras*^G12D^ in all thymic cells (including lymphocytes) suggested that recombination could occur independently from *Pdx1* expression. Indeed, in the original study using *Kras*^G12D^ oncogene in mouse model, Johnson and colleagues studied the organs, which were sensitive to sporadic recombination (unequal sister chromatid exchange or intra-chromosomal recombination) leading to *Kras*^G12D^ expression. Interestingly, they highlighted the development of lung tumors, thymic lymphomas and papillomas, suggesting that lung, thymus and skin were the organs in which sporadic recombination occurred^41^. The development of thymic masses we observed could thus be relied on sporadic recombination. In addition, we frequently observed papillomas on KC mice, with no deleterious effect on animal well-being (data not shown), coherently with the study of Johnson and collaborators. In addition, we highlighted that three out of the four mice developing thymic mass exhibited some malignancies in lung, more or less visible, that were positive for KRAS^G12D^ and highly proliferative, as expected in consequence to KRAS activation. The mouse showing no lung disorder was dissected as scheduled, at 100 days, and had not reached humane end-points, suggesting that lung mass development could arise later. Lung being a known metastasis site for pancreatic tumor cells, these malignancies could be metastases, but the hypothesize seems to be ruled out by the fact that they are clearly negative for CK19 and do not display PDAC morphology. In addition, no correlation existed between pancreatic lesion extent and level of lung damage. In regards with the literature, it appears that such lung malignancies could be explained by sporadic recombination, as pointed out by Johnson and colleagues in 2001, who described lung cancer development as a consequence of *Kras*^G12D^ expression only mediated by sporadic recombination (no CRE)^41^.

Therefore, we suggest here that *Pdx1*-CRE construct is not sufficient to restrain *Kras*^G12D^ recombination to the pancreas, and that sporadic recombination occurs in thymus (in 10% of cases) and lung. This is coherent with the fact that no evidence of *Pdx1* expression in the thymus has been found, and some articles even reported the absence of Pdx1 in the thymus^42,43^. However, *Pdx1* has already been shown to be expressed in skin, linked to the development of papillomas in KC mice^44^. To finally confirm our hypothesis, it would be interesting to perform Pdx1 labeling on thymus and thymic masses, which will allow to discriminate between sporadic or *Pdx1*-induced recombination. However, *Pdx1* expression in thymic cells would probably be transient, and could thus be difficult to highlight. In another hand, (1) thymocytes are the place of many genetic recombination (Variable (Diversity) Junction (V(D)J) recombination), leading to a large panel of variable regions on T cell receptors^45^, and (2) sporadic recombination is favored by the presence of repeated sequences^46,47^, as can be considered Lox sequences surrounding transcriptional STOP upstream *Kras*^G12D^ allele added by Jackson and colleagues in 2001^24^. Moreover, lymphocyte population has recently been shown to be sensitive to Kras activation^48^. All these considerations thus encourage the sporadic recombination hypothesis.

The KC mouse model is one of the most used models to study pancreatic carcinogenesis. The cohort we initially developed (89 mice) aimed at determining the role of a matrix glycoprotein during pancreatic carcinogenesis, study which was particularly impacted by the development of the adverse effect we describe here. By investigating the impact of thymic mass development on pancreatic carcinogenesis, we unfortunately highlighted that thymic tumor occurrence was associated with an accelerated tumorigenesis. Indeed, we noted that (1) three out of the four analyzed mice suffering from thymic tumor displayed a larger extent of pancreatic lesions, even if this result was non-significant and (2) those lesions significantly presented an increased proliferation, as assessed by Ki67-positive cell quantification. Altogether, those results confirm that mice exhibiting thymic mass presented higher grade pre-tumoral to tumoral lesions, and thus an accelerated pancreatic carcinogenesis. In addition, we detected some KRAS^G12D^-positive cells within the adjacent spleen, suggesting an invasive phenotype for pancreatic (pre)tumoral cells (Supplementary Figure S1). Thus, the presence of thymic tumor affects the study of the pancreatic carcinogenesis process.

The accelerated carcinogenesis could be due to immune disorder within these mice. This hypothesis is corroborated by (1) the disorganization we observed within the spleen, with the perturbation of white pulp structure, and (2) high level of immune infiltration within the pancreas of two out of the mice with thymic mass, as demonstrated by CD3e immunolabeling. Indeed, disorder in thymus could affect T cell compartment and lead to the absence or the inefficiency of tumor-suppressing immune response in first steps of pancreatic carcinogenesis^49^. In addition, in the mouse presenting high level of immune infiltration, it was interesting to note that lymph nodes and immune cells around pancreatic cells were positive for KRAS^G12D^ protein, as well as in spleen white pulp, suggesting either (1) sporadic recombination or (2) the recruitment of immune cells from thymic mass, hypothesis that we favor. Finally, the labeling of KRAS^G12D^ was also quite interesting to understand the appearance of lesions in pancreas of KC mice. Indeed, we detected KRAS^G12D^ within preneoplastic lesions, as well as in Langerhans islets, as expected by the expression of *Pdx1* in these structures^25,33,34^. The presence of KRAS^G12D^ in the islets could explain the islet disorganization, as well as the lesions developing within islets that have been previously described^35,36^. Interestingly, we also highlighted KRAS^G12D^ in acinar cells surrounding pre-tumoral lesions and evoking first steps of ADM (less contiguous acini and with more visible light), suggesting that *Pdx1* expression could be induced by the lesion and lead to transformation of adjacent normal acinar cells.

Our study thus pointed out an adverse effect of the *Kras*^*G12D*^ construct in the KC mouse model classically used to study pancreatic carcinogenesis. To our knowledge, this is the first report showing the development of such thymic tumors in the KC mouse model. The hypothesis of sporadic recombination in organs with high level of recombination (thymus notably) is favored here. This bias is associated with an accelerated pancreatic carcinogenesis, and can thus lead to misinterpretations, knowing that thymic mass can be missed if only the pancreas is recovered (without opening the rib cage).

## Methods

### Animals

LSL:*Kra*s^G12D^ (B6.129S4-*Kras*^*tm*4*Tyj*^/J strain, The Jackson Laboratory) and *Pdx1*-CRE (B6.FVB-Tg(Pdx1-cre)6Tuv/J strain, The Jackson Laboratory) mouse strains were previously described^24,25,41^ and generously gifted by P. Bertolino and L. Bartholin, respectively. They were maintained in two pathogen-free animal facilities (‘‘ALECS-SPF” and “PBES” (Lyon, France)), and crossed in order to obtain [LSL:*Kras*^*G12D*^;*Pdx1*-CRE] mice, predisposed to pancreatic cancer development. All procedures were conducted in accordance with the guidelines of the European Union and French laws and approved by the local animal ethic committee under regulatory of governmental authority (CECCAPP, Comité d'Evaluation Commun au Centre Léon Bérard, à l'Animalerie de transit de l'ENS, au PBES et au laboratoire P4 (n° C2EA15), APAFIS #16,242–201,804,271,428,498). The study was carried out in compliance with the ARRIVE guidelines (Animal Research : Reporting of In Vivo Experiments)^50^. Animals were genotyped one week after birth and body weight tracking was started at 100 days post-parturition and was performed twice a week. Mice were euthanized either at 100-, 150- or 200-days post-parturition or at reaching end-point (weight loss, signs of pain, etc.), and cumulative survival percentage was estimated using the Kaplan–Meier method, before comparing groups thanks to Log-Rank (Mantel-Cox) statistical test. At necropsy, several organs were harvested, including pancreas, spleen, lung, thymus, as well as the tail for control. After dissection, one part of each organ (except tail) was fixed in formalin before being processed for paraffin embedding. The other part was directly snap frozen in liquid nitrogen for DNA extraction.

### Histological staining and immunohistochemistry

For histological analyses, formalin-fixed paraffin embedded (FFPE) tissues were cut into 3 µm sections and Hematoxylin and Eosin (H&E) staining was performed after dewaxing and rehydration.

Immunohistochemistry on FFPE tissues was performed on 3 µm sections using ImmPRESS Excel Amplified Polymer Staining Kit, Anti-Rabbit IgG, Peroxidase (VECTOR LABORATORIES, MP-7601) for Ki67, CD3e and KRAS^G12D^, and R.T.U. Vectastain Universal Elite ABC Kit (VECTOR LABORATORIES, PK-7200) for CK19, following manufacturer’s instructions. After deparaffinization and rehydration, antigen retrieval was performed either in Tris–EDTA buffer (pH9) (CD3e) or Sodium Citrate buffer (pH 6) (Ki67 and CK19) for 20 min at 98 °C followed by 20 min cooling, or in pepsin reagent (Sigma, R2283) for 30 min at 37 °C (KRAS^G12D^). Then, endogenous peroxidases were quenched and slices were saturated with 2.5% to 10% Horse Serum. Primary antibodies (CK19 (1/100, #TROMA-III-s, DHSB); Ki67 (prediluted, #RMPD-004, CLINISCIENCES); KRAS^G12D^ (1/50, #14,429, CELL SIGNALING); CD3e (1/500, MA5-14,524, THERMOFISHER)) were incubated in blocking solution or 1% Horse Serum (KRAS^G12D^) overnight at 4 °C. The next day, amplification and development were performed following manufacturer’s instructions, nuclei were counterstained with Gill’s Hematoxylin and slices were mounted in DEPEX. All slices were imaged using the slide scanner AxioScanSP5 X (ZEISS) at CIQLE (Lyon, France) in order to have a general view of all tissues. Representative images were then extracted and shown. Quantification of lesion extent and proliferation was performed thanks to QuPath software, by calculating area of lesions (H&E staining) and proportion of proliferating cells (Ki67-positive nuclei), respectively. Groups were compared thanks to Mann–Whitney statistical test (non-parametric* t* test).

### *Kras*^*G12D*^ PCR

Genomic DNA extraction was performed by incubating tissues for 30 min in alkaline lysis solution (25 mM NaOH, 0.2 mM Na_2_EDTA) at 95 °C. Tissue lysis was stopped by adding neutralization solution (40 mM Tris–HCl) (v/v) on ice.

[LSL:*Kras*^*G12D*^;*Pdx1*-CRE] mice were genotyped by PCR amplification of genomic DNA from tail, as advised for this mouse strain ("https://www.jax.org/Protocol?stockNumber=008179&protocolID=29388"). PCR mix was composed of 1X TAQ polymerase buffer, 2.5 mM MgCl2, 1 mM dNTP (each), 1 µM primers (each), 1U TAQ Polymerase. Primers used were: (p1) 5′ TGTCTTTCCCCAGCAGAGT 3′, (p2) 5′ CTGCATAGTACGCTATACCCTGT 3′, (p3) 5′ GCAGGTCGAGGGACCTAATA 3′. 2 µl of DNA was added to the mix and PCR was performed following cycles: 5 min at 96 °C, 35 cycles (30 s at 96 °C, 35 s at 60 °C, 45 s at 72 °C), 5 min at 72 °C. PCR amplicons were separated on a 2% (w/v) agarose gel in order to detect the 250 bp wild-type *Kras* and 100 bp floxed LSL:*Kras*^*G12D*^ products.

*Kras*^*G12D*^ conditional PCR was performed on thymus, pancreas and tail genomic DNA as mentioned by The Jackson laboratories ("https://jacks-lab.mit.edu/protocols/genotyping/kras_cond"). PCR mix was composed of 1X TAQ polymerase buffer, 2.5 mM MgCl2, 0.4 mM dNTP (each), 0.4 µM primers (each), 1U TAQ Polymerase. Primers used were: (p1) 5′ GTCTTTCCCCAGCACAGTGC 3′, (p2) 5′ CTCTTGCCTACGCCACCAGCTC 3′, (p3) 5′ AGCTAGCCACCATGGCTTGAGTAAGTCTGCA 3′. 1 µl of DNA was added to the mix and PCR was performed following cycles: 5 min at 96 °C, 35 cycles (30 s at 96 °C, 30 s at 63 °C, 45 s at 72 °C), 5 min at 72 °C. PCR amplicons were separated on a 2% (w/v) agarose gel in order to detect the 622 bp wild-type *Kras,* 500 bp floxed LSL:*Kras*^*G12D*^ and 650 bp recombined *Kras*^*G12D*^ products. Position of primers and expected results are presented in Fig. 1a.

## Supplementary Information

Supplementary Information.
