# Supplementary material for: Development of thymic tumor in [LSL:KrasG12D; Pdx1-CRE] mice, an adverse effect associated with accelerated pancreatic carcinogenesis
Source: Sci Rep. 2021 Jul 23;11:15075. doi: 10.1038/s41598-021-94566-x (PMC8302691; doi:10.1038/s41598-021-94566-x)
Supplement: Supplementary file 1 — Supplementary Information. [file 41598_2021_94566_MOESM1_ESM.pdf]

Supplementary data

Development of thymic tumor in [*LSL:Kras*^{G12D}; *Pdx1-CRE*] mice, an adverse effect associated with accelerated pancreatic carcinogenesis.

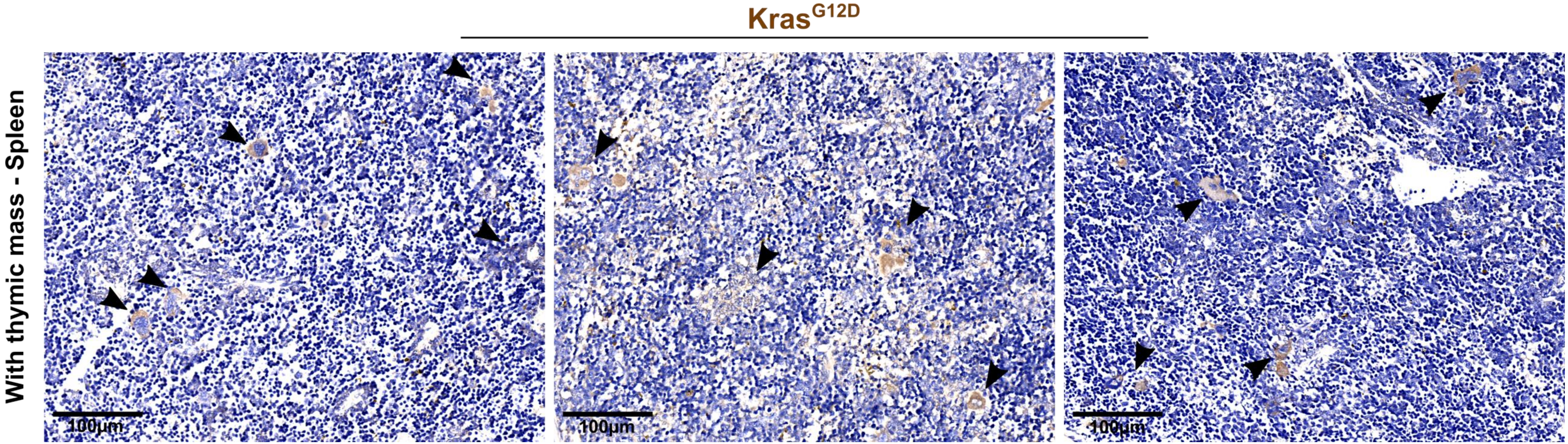

Supplementary Figure S1: Presence of KrasG12D positive cells in spleen adjacent to pancreas. *Representative images of spleen adjacent to pancreas, labelled through anti-KRAS^{G12D} immunohistochemistry. Kras^{G12D} positive cells evoking invasion from pancreas to spleen are pointed by arrowheads. Scale bars are indicated on the pictures.*

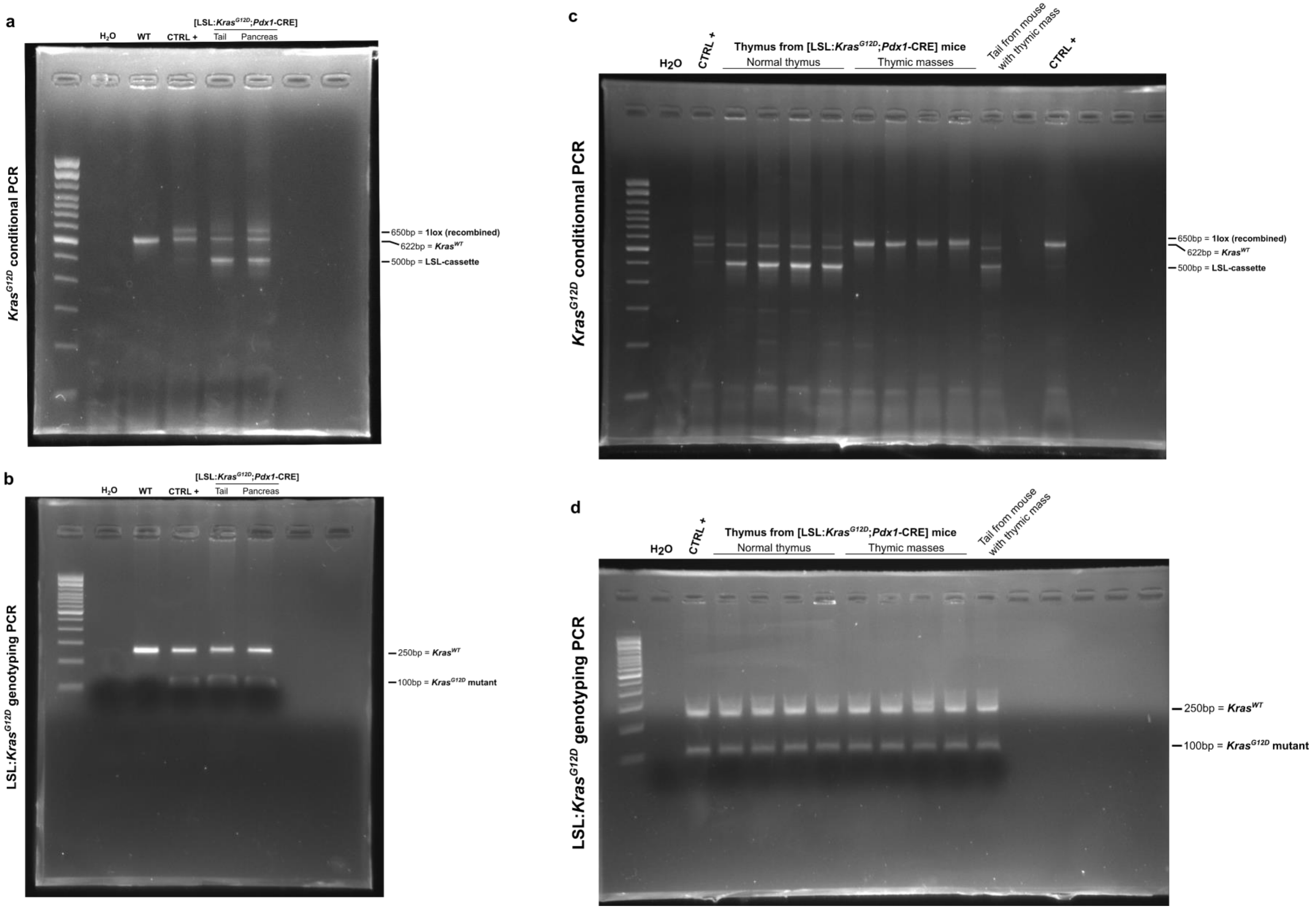

Supplementary Figure S2: Uncropped pictures of the gels presented *in Figure 1b Top (**a**), Figure 1b Bottom (**b**), Figure 3b Top (**c**) and Figure 3b Bottom (**d**).*
